# Upregulation of TMEM40 is associated with the malignant behavior and promotes tumor progression in cervical cancer

**DOI:** 10.1007/s12672-023-00648-9

**Published:** 2023-04-13

**Authors:** Zhen-Fei Zhang, Fang Liu, Han-Rong Zhang, Bing Liu, Shu-Qian Zheng, Wan-Qian Ye, Jia-Nan Ding, Ze-Jie Zhou, Hui-Xian Luo, Fang Wu, Xuan-Min Guo, Jue-Yu Zhou, Yong-Hui Guo

**Affiliations:** 1 Department of Laboratory Medicine, Zhujiang Hospital, Southern Medical University, 253 Industrial Avenue Central, Guangzhou, 510280 People’s Republic of China; 2 Department of Biochemistry and Molecular Biology, School of Basic Medical Sciences, Southern Medical University, Guangzhou, 510515 People’s Republic of China; 3 Department of Nursing and Health, Nanfang College-Guangzhou, Guangzhou, 510970 Guangdong People’s Republic of China; 4 Department of Urology, Zhujiang Hospital, Southern Medical University, Guangzhou, 510515 People’s Republic of China

**Keywords:** TMEM40, Cervical cancer, Malignant phenotype, Tumor progression, p53 pathway

## Abstract

**Objective:**

Recent studies indicated that transmembrane protein 40 (TMEM40) is associated with several types of cancers but is not clear in cervical cancer (CC). The study aimed to examine the role of TMEM40 in CC and related mechanisms.

**Methods:**

The expression of TMEM40 in CC tissues and cell lines was studied with western blot and real-time quantitative RT-PCR. The effect of TMEM40 on proliferation was evaluated by CCK-8, EdU and colony formation assay. The migration, invasion, cell cycle and apoptosis of CC cells were studied with wound healing, transwell assays and flow cytometry. Tumor growth was evaluated in vivo using a xenogenous subcutaneously implant model.

**Results:**

The results revealed that the TMEM40 elevation in CC tissues and cell lines was closely correlated with tumor size and lymph node metastasis in clinical patients. Upregulation of TMEM40 with OE-TMEM40 vector promoted the invasion, migration and proliferation, inhibited the apoptosis and led to distinct S cell cycle arrest in CC cell lines. Silencing TMEM40 with shRNA inhibited the invasion, migration and proliferation, promoted apoptosis and led to a G0/G1 cell cycle arrest in CC cell lines. Silence of TMEM40 downregulated the expression of c-MYC, Cyclin D1, matrix metalloproteinase-1 (MMP-1) and matrix metalloproteinase-9 (MMP-9), but in contrast, activated p53 and several apoptosis related proteins such as p53, Caspase-3, Caspase-9 and PARP1. In addition, TMEM40 silencing dramatically decreased tumor growth in mice models.

**Conclusion:**

The present study demonstrates that TMEM40 upregulation can be a potential prognostic biomarker and contribute to CC development.

## Introduction

Cervical cancer (CC) is a common gynecologic malignant tumor. Most cases of CC are caused by infection with high-risk types of human papillomavirus (hrHPV) [1]. Despite population-based studies showing that vaccination against human papillomavirus reduces the incidence of cervical pre-cancer [2]. Human papillomavirus vaccine coverage is inadequate worldwide, especially in low-income countries where the disease burden is highest [3]. Therefore, morbidity and mortality rates are high in women, with approximately 350,000 women dying from CC each year [4, 5]. Lymph node metastasis, recurrence, and distant metastasis still occur in about 30% of patients after initial treatment for CC [6], which is the major cause of treatment failures for most patients. Although there is a great reduction in the mortality of CC in recent years, the underlying molecular mechanisms of CC metastasis remain unclear.

Transmembrane protein 40 (TMEM40), encoded by the *TMEM40* gene and localized to chromosome 3p25. It is a multichannel membrane protein containing 233 amino acids [5, 6]. TMEM40 exists in two subtypes and has previously been reported to play an important role in collagen-induced arthritis (CIA) [7–9]. The prevention and treatment of CC are urgent. Our previous finding suggested that the expression of TMEM40 in bladder cancer was significantly related to the pathologic grade, clinical stage, histological grade and pT status of bladder cancer [10]. Moreover, recent studies have reported that lower TMEM40 expression is associated with better overall survival in cervical cancer, suggesting that TMEM40 is a potential prognostic indicator [11]. Given the role of TMEM40 in bladder cancer, it is important to clarify the abundance of TMEM40 expression in CC and whether it can play a role in the regulation of cell malignant behavior in CC, as well as its unknown regulatory molecular mechanism.

In this study, we identified correlations between TMEM40 expression in CC cells and clinicopathological features at the protein and mRNA levels. These data provide powerful evidence for the role of TMEM40 in promoting the progression of CC and suggest that TMEM40 may be a potential therapeutic target for patients with CC.

## Methods

### Patients and tissue specimens

The fresh CC tissues with non-tumor tissue samples were selected from 15 CC patients who did not receive preoperative treatment and were treated with surgical intervention from February 2019 to July 2020. All the tissues were obtained with written informed consent of patients. The Ethics Committee of the ZhuJiang Hospital of Southern Medical University approved the utilization of these CC samples for research purposes only and all the experiments in the study.

### Cell lines and cell culture

The human cervical cancer cell lines (C33A, SiHa, CaSki and HeLa) were purchased from the Culture Collection of the Chinese Academy of Sciences, Shanghai, China. The CaSki and SiHa cells were cultured in high glucose DMEM (Life Technologies, Shanghai, China), the C33A and HeLa cells were grown in RPMI-1640 (Life Technologies, Shanghai, China), both medium were supplied with 10% fetal bovine serum (FBS, Gibco, USA) and 1% ampicillin and streptomycin (100 units/mL, Life Technologies, Shanghai, China), at 37 °C with a humidified atmosphere of 95% air and 5% CO_2_ in incubator.

### Plasmid construction and cell transfection

The full-length complementary cDNA of human TMEM40 was synthesized by Invitrogen and cloned into the expression vector pEZ-M98 (Takara Bio USA, Inc.). The small hairpin RNA (shRNA) of the TMEM40 was provided by Invitrogen Corporation (Grand Island, NY, USA), and the final construct was verified by sequencing. Plasmid vectors for transfection were prepared using DNA Midiprep Kits (Qiagen, Hilden, Germany) and transfected into CC cells. The shRNAs were transfected into CC cells using Lipofectamine 2000 (Invitrogen) according the manufacturer’s instructions. The sequence of shRNA was 5ʹ-GUGGACGCCUCUCAG UUAA-3′; NC was 5′-TTCTCCGAACGTGTCACGT-3′.

### Western blot

The expression of TMEM40 protein in CC tissues and cell lines were evaluated with western blot. Briefly, the CC tissues and cells were lysed, and proteins were extracted through standard protocols. The proteins were separated by SDS–polyacrylamide gel electrophoresis and were transferred to polyvinylidene difluoride (PVDF) membranes. The PVDF membrane was blocked with 5% bovine serum albumin for 2 h. The immunoblots were incubated overnight at 4 °C with the following primary antibodies: mouse anti-TMEM40 (1:1000, Santa Cruz, CA, USA), rabbit anti-p53, rabbit anti-p21, mouse anti-CCND1, rabbit anti-c-MYC, rabbit anti-PARP1, rabbit anti-Caspase-9, rabbit anti-Caspase-3, rabbit anti-MMP1 and rabbit anti-MMP9 (1: 1000, Proteintech Group, INC, USA). The membranes were washed, incubated with appropriate HRP-conjugated secondary antibodies at room temperature, 2 h), and detected using the enhanced chemiluminescence detection system. GAPDH (1: 8000, CST, Inc, MA, USA) was used as a loading control.

### RNA isolation and real-time quantitative RT-PCR analysis

Total RNA was isolated by using Trizol reagent (Invitrogen, Carlsbad, CA, USA) according to the manufacturer’s instructions. Reverse transcription and RT-qPCR kits (Takara Bio, Inc.) were used to evaluate the expression of TMEM40 mRNA. GAPDH was used as a control to normalize the expression of TMEM40 protein. The qRT-PCR primers and their sequences were listed in Table 1.

**Table 1:** Relationship of TMEM40 expression between cervical cancer tissues and para-carcinoma tissues

TMEM40 staining				
	All cases (100%)	Negative expression(%)	Positive expression(%)	P value^†^
Para-carcinoma tissues	31	20(64.5%)	11(35.5%)	0.002
Cervical tumor tissues	31	8(25.8%)	23(74.2%)	

^†^*P* value are from Chi-square test

### Cell proliferation assay

The cell viability was determined using the CCK-8 assay. Briefly, 2000 cells/well were seeded into 96-well plates in a volume of 100 μL complete medium containing 10% FBS. At 0, 24, 48, 72, and 96 h after transfection, the cells were incubated with CCK8 reagent (Beyotime Inst Biotech, China) at 37 °C for 2 h and the absorbance at 450 nm was measured using a microplate reader (Tecan, Infinite^®^M200, Austria). Data were collected from three separate experiments with four replications each time.

### Plate colony formation assay

The CC cell lines of CaSki and SiHa were suspended in DMEM containing 10% FBS. A total of 500 cells were then seeded into each well of a 6-well plate. The cells were incubated at 37 °C for 14 days with growth media being replaced every third day. After 14 days of cultivation, the colonies were stained with 0.5% crystal violet (Beyotime Inst Biotech, China) and counted.

### Wound healing assay

The cells were cultured in 6-well plates, and, when the confluence reached 90%, the cell layers were scratched using a 10 μL tip to form wounded gaps, washed with PBS twice and cultured. The wounded gaps were photographed at different time points and analyzed by measuring the distance of migrating cells from five different areas for each wound.

### Transwell migration and invasion assays

The cell migration and invasion were evaluated using a transwell assay (8 μm pore size, BD Biosciences, New Jersey, USA). The bottom well contained growth medium with 20% FBS while the top chamber contained 200 μL of serum-free medium with a total of 2 × 10^4^ cells transfected with shRNA or OE-TMEM40 vector 24 h early and the cells were incubated at 37 °C for 24 h. Then, the cells that migrated to the lower face of the filters were fixed with 100% methanol and stained with 0.5% crystal violet. The invaded cells were counted in three randomly selected fields under a microscope, and the average was calculated. Each experiment was conducted in triplicate.

### Cell cycle analysis

The cells were detached using trypsinization, washed twice with precooled PBS, and fixed in 70% ethanol at − 20 °C overnight. The fixed cells were suspended in 50 μL of RNaseA (KeyGEN Biotech, Nanjing, China) and 450 μL of propidium iodide (PI) (KeyGEN Biotech, Nanjing, China), and incubated at room temperature for 30 min in dark. After filtration, the cell cycle was examined by flow cytometry.

### Apoptosis assessment

Following treatment, the cells were washed with PBS and then stained using the Annexin V-FITC Apoptosis Detection Kit (KeyGEN Biotech, Nanjing, China) according to the according instruction. The cells were analyzed with a flow cytometer (San Jose, CA, USA).

### In vivo xenograft study

All the animal procedures were performed by the institutional ethical guidelines and approved by the Experimental Animal Ethics Committee of the Southern Medical University. SiHa cells (1 × 10^7^) with stably transfected shRNA-TMEM40 or shRNA-NC were suspended in 0.2 mL PBS and injected into the left flank of 5-week-old female BALB/c athymic nude mice. Tumor volumes were calculated using hand calipers every 3 days after the injection using the following formula: tumor volume (mm^3^) = (L × W^2^)/2. At 32 days, the mice were sacrificed and tumor volumes and weights were recorded.

### Statistical analysis

The data were expressed as mean ± SD and analyzed with GraphPad Prism 5 (La Jolla, CA, USA). The differences were examined using paired or unpaired Student’s t-test, a one-way ANOVA and χ^2^ tests. A p-value of < 0.05 was considered statistically significant.

## Results

### TMEM40 expression was increased in cervical cancer tissues and CC cell lines

**Fig. 1:**
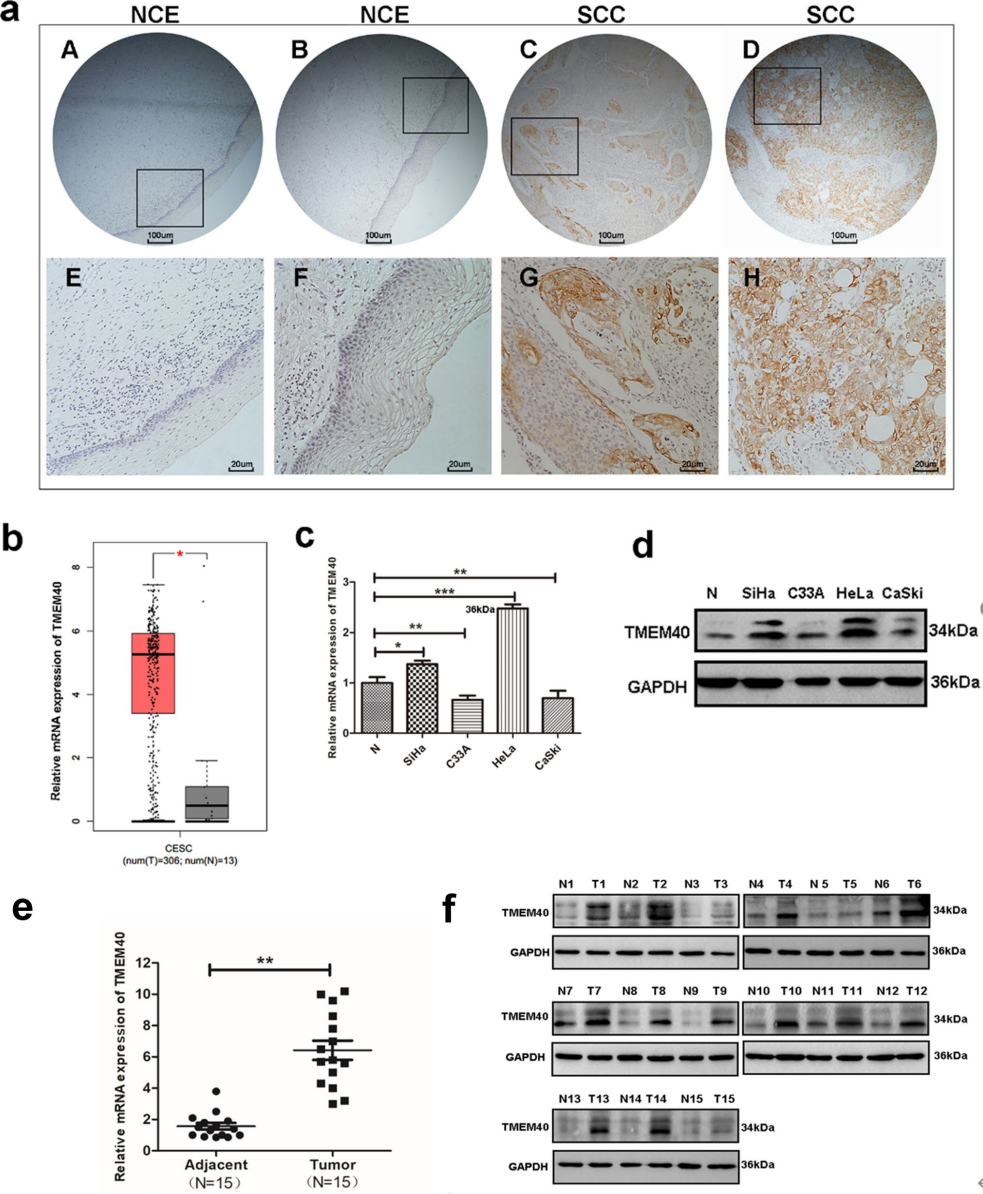
TMEM40 expression level is significantly upregulated in cervical cancer tissues and cell lines. **a**, Representative immunohistochemical staining of TMEM40 protein in normal cervical epithelium (NCE) and cervical squamous cell carcinoma (SCC) with different staining intensities (A–D, 40 × ; E–H, 200 ×). Lower panels (200 ×) were high magnification of squared areas in upper panels (40 ×). **b**, Quantification of TMEM40 mRNA expression in CC and normal tissues. TMEM40 expression was significantly increased in CC tissues (n = 306) compared with normal tissues from the patients (n = 13) from the GEPIA dataset. **c** and **d**, qRT-PCR and western blot analysis of TMEM40 mRNA and protein expression in CC cell lines (C33A, HeLa, SiHa and CaSki) and normal cervical epithelium (N). **e** and **f**, Expression level of TMEM40 in tissues was detected by qRT-PCR and western blot. *P < 0.05, **P < 0.01, ***P < 0.001

The expression of TMEM40 in CC tissues was investigated with IHC and the results indicated that TMEM40 expression varied from normal cervical to cervical cancer tissues (Fig. 1a, Table 2). Analysis of Gene Expression Profiling Interactive Analysis (GEPIA) site suggested that TMEM40 levels in tumor tissue (N = 306) were observably higher than those in normal cervical tissue (N = 13) (Fig. 1b). Meanwhile, the expression of TMEM40 in CC cell lines (i.e. C33A, CaSki, SiHa and HeLa), one normal uroepithelial tissue (Fig. 1c and d) and 15 fresh CC tissues paired with their adjacent non-neoplastic cervical tissues (Fig. 1e and f) was determined by quantitative RT-PCR and western blot analysis. As shown in Table 3, high expression of TMEM40 was markedly associated with lymph node metastasis (p = 0.010) and tumor size (p = 0.006). But, no significant associations were found between TMEM40 level and age, pathological grade, and pT Status (all at p > 0.05, respectively). The results indicated that TMEM40 expression was upregulated in CC cell lines and tissues.

**Table 2:** Relationship of TMEM40 expression and clinicopathological features in cervical cancers

Number of patients		TMEM40 protein expression		
		Negative	Positive	P value^b^
All patients	31	8	23	
Age(years)				
≤ 48^a^	14	3(21.4%)	11(78.6%)	0.613
> 48	17	5(29.4%)	12(70.6%)	
Lymph node metastasis				
No	15	7(46.7%)	8(53.3%)	0.010
Yes	16	1(6.3%)	15(93.7%)	
Tumor size(cm)				
< 3	8	5(62.5%)	3(37.5%)	0.006
≥ 3	23	3(13.1%)	20(86.9%)	
Pathological grade				
I	19	6(31.6%)	13(68.4)	0.355
II-III	12	2(16.7%)	10(83.3%)	
pT Status				
T1	13	4(30.8%)	9(69.2%)	0.592
T2-T3	18	4(22.2%)	14(77.8%)	

^a^ Mean age. ^b^ *P* value are from Chi-square test

**Table 3:** The primers used in qRT-PCR analysis

Gene	Sense and antisense primer (5ʹ-3ʹ)
TMEM40	F: GCGGTAGGGGTGTACGGT
	R: CCGGACACGCTGAACTTGT
P53	F: AAGATCCGCGGGCGTAA
	R: CATCCTTTAACTCTAAGGCCTCATTC
Rb	F: ATCAAGGGTCATTATGGGTTAGGC
	R: AGGTGTAGGGGAGGGGAGAAGC
JNK2	F: TACGTGGTGACACGGTACTACC
	R: CACAACCTTTCACCAGCTCTCC
C-MYC	F: CTTCTCTCCGTCCTCGGATTCT
	R: GAAGGTGATCCAGACTCTGACCTT
MMP1	F: GCGCACAAATCCCTTCTACC
	R: ATCCGTGTAGCACATTCTGTCC
MMP9	F: TTGGTCCACCTGGTTCAACT
	R: CGACGTCTTCCAGTACCGA
GAPDH	F: CAGCCTCAAGATCATCAGCA
	R: TGTGGTCATGAGTCCTTCCA

### Downregulation of TMEM40 inhibited CC cell proliferation, migration, invasion and promoted cell apoptosis in vitro

**Fig. 2:**
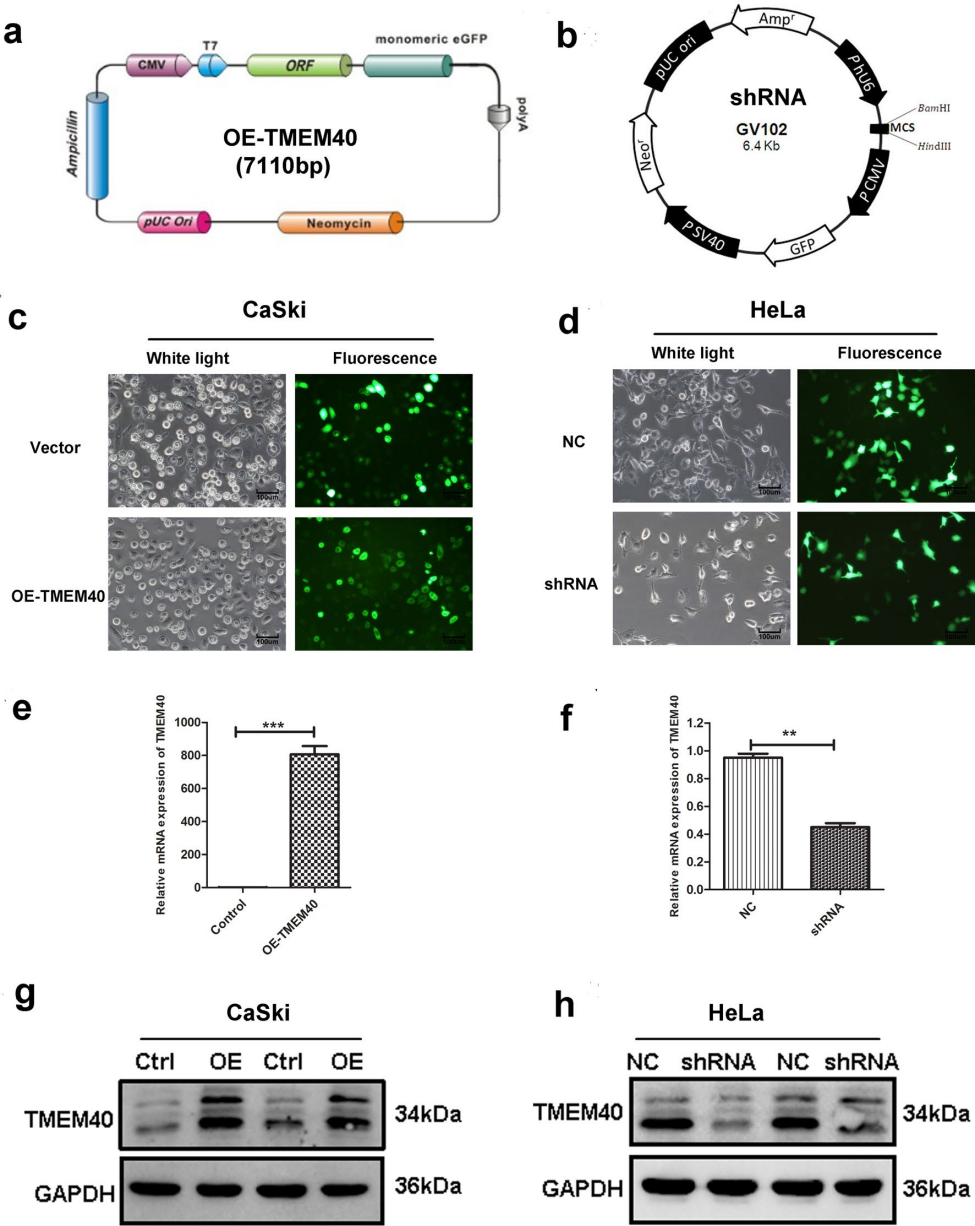
OE-TMEM40 and shRNA vector construction and the expression of TMEM40. **a** and **b**, The OE-TMEM40 and TMEM40 shRNA vector was engineered to express TMEM40 under the T7 and U6 promoter. **c** and **d**, The expression of green fluorescent protein after 48 h of OE-TMEM40 and TMEM40 shRNA vector transfection in CaSki and HeLa cells (magnification × 100). **e** and **g**, The OE-TMEM40 significantly upregulated the expression level of TMEM40 in CaSki cells. **f** and **h**, The TMEM40 shRNA vector significantly down-regulated the expression level of TMEM40 in HeLa cell. **P < 0.01, ***P < 0.001

To investigate the functions of TMEM40 in CC, TMEM40 shRNA and OE-TMEME40 plasmid containing green fluorescence protein (GFP) were used to decrease or increase the expression of TMEM40 in CC cells. Then fluorescence intensity was observed under fluorescence microscope to evaluate plasmid transfection efficiency (Fig. 2a–d). The data displayed that overexpression of TMEM40, mRNA (Fig. 2e) and protein (Fig. 2g) levels were up-regulated in CaSki cells, while TEME40 shRNA significantly reduced TMEM40 expression in HeLa cells (Fig. 2f, h).

**Fig. 3:**
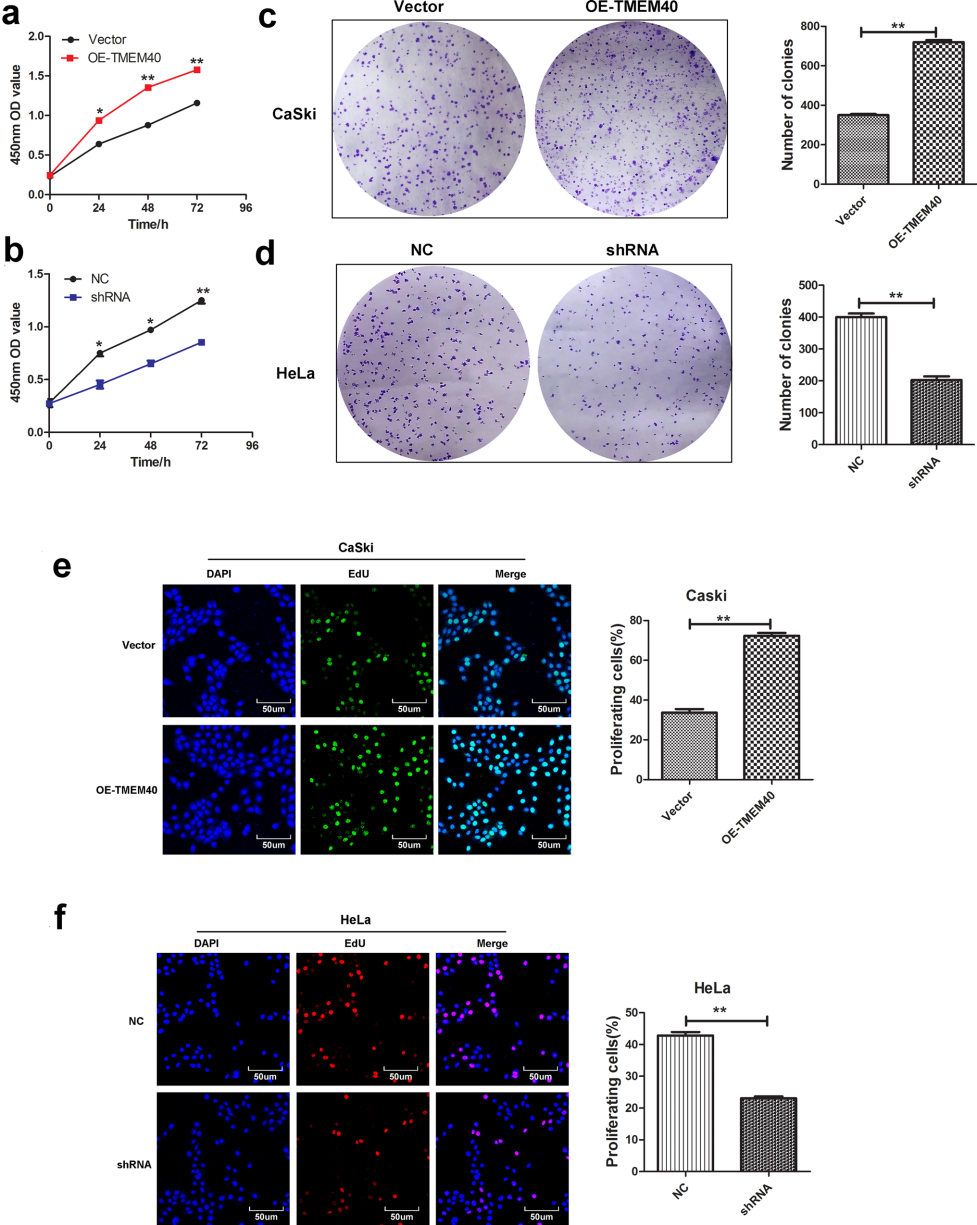
Knockdown of TMEM40 expression suppresses the viability of cervical cancer cells. **a** and **b**, Growth curves of CaSki and HeLa cells transfected with OE-TMEM40 or TMEM40 shRNA vector. Cell growth was determined by CCK8.** c** and **d,** A Colony-formation assay was performed to determine the oncogenic growth of CaSki and HeLa cells. **e** and **f**, EdU assay was performed to examine the proliferation of CaSki and HeLa cells. *P < 0.05, **P < 0.01

**Fig. 4:**
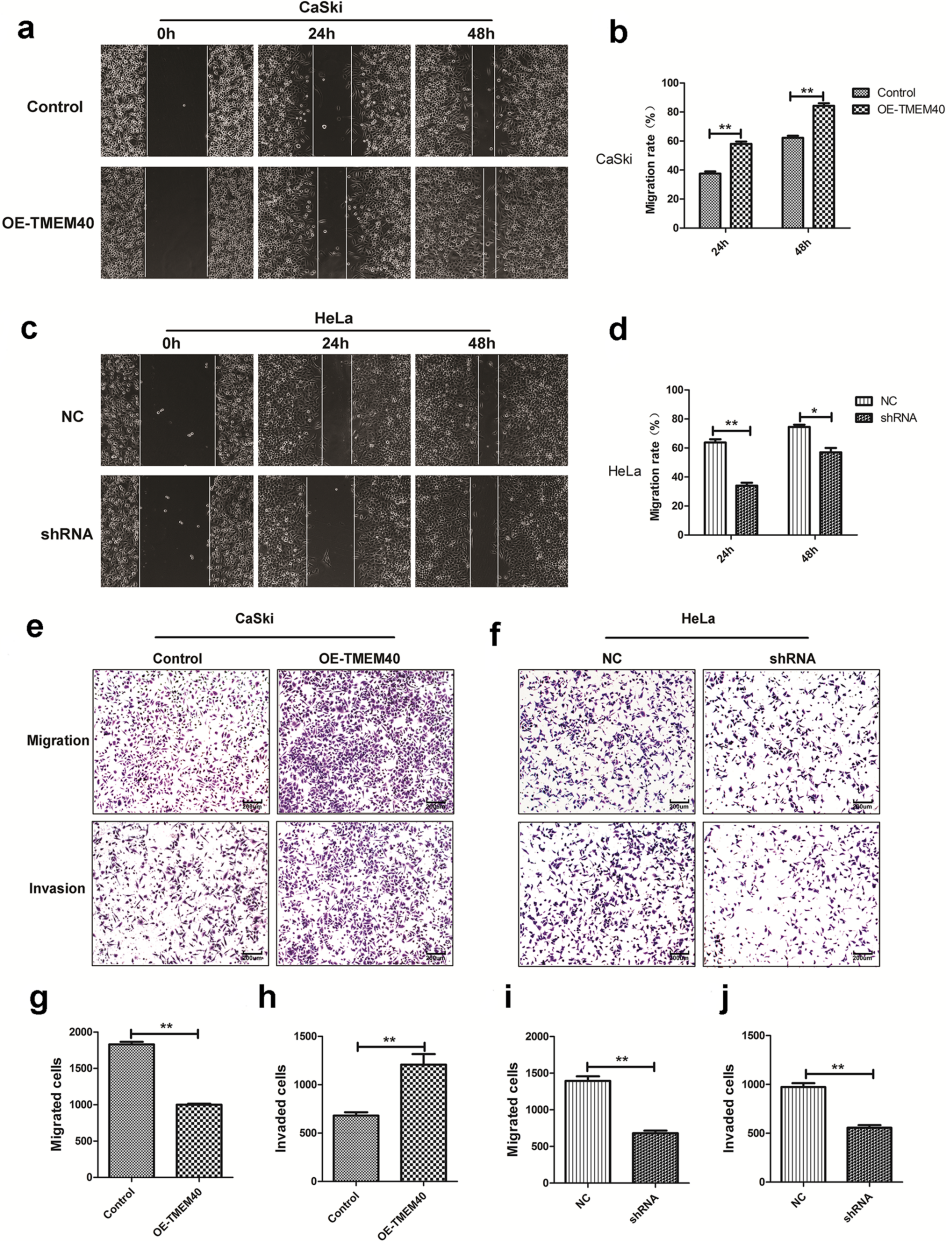
TMEM40 regulates CC cell migration and invasion in vitro. **a-b** and **c-d**, Wound healing assays were performed to determine the migratory abilities of OE-TMEM40-transfected CaSki cell and TMEM40 shRNA transfected HeLa cell, respectively. **e-j**, Transwell assays with matrigel were performed to determine the migratory and invasive abilities of OE-TMEM40 or shRNA transfected CaSki and HeLa cells. *P < 0.05, **P < 0.01

**Fig. 5:**
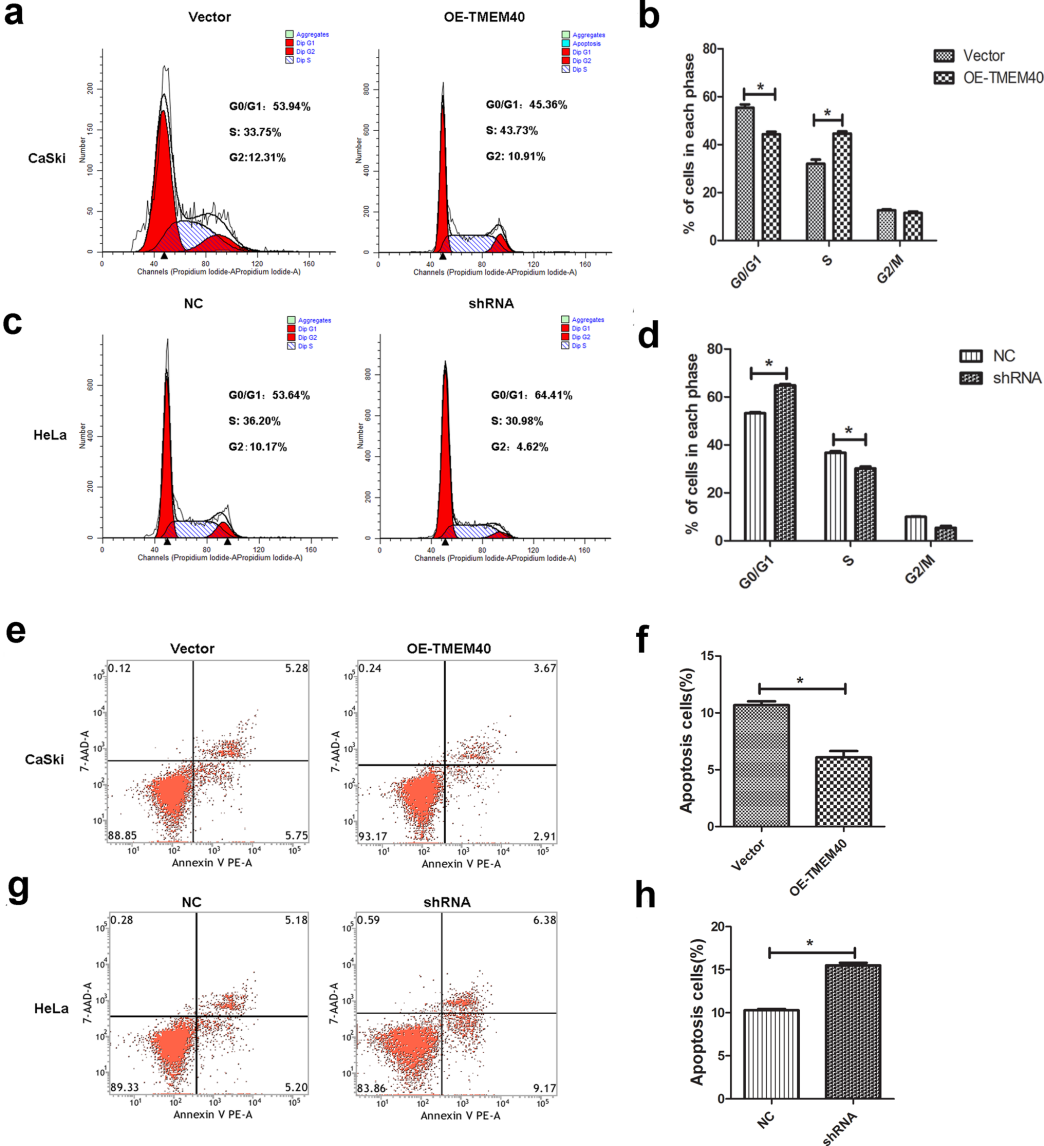
TMEM40 regulates CC cell cycles and apoptosis in vitro. **a-b** and** c-d**, Cell cycle analysis determined the relative cell numbers in each cell-cycle phase after propidium iodide staining of OE-TMEM40 or TMEM40 shRNA transfected CaSki and HeLa cells. **e**–**f** and **g**-**h**, Flow cytometry assays were performed to determine apoptotic cells after 48 h transfection with OE-TMEM40 or TMEM40 shRNA compared to their controls. *P < 0.05

Next, we used CCK-8, plate colony formation and EdU assays to assess the effect of TMEM40 on CC cells growth. The growth curves suggested that inhibition of TMEM40 significantly decreased cell proliferation ability compared with the control group (Fig. 3a, b). As expected, the number of colonies and EdU fluorescence of the HeLa cells with TMEM40 knockdown were significantly lower than those of shNC control cells (Fig. 3c−f). Furthermore, wound healing and transwell assays showed that there was significantly slower closing of scratch wounds and fewer migrated cells in the shRNA-TMEM40 group than in the shNC control cells (Fig. 4a–j). Based on the effect of TMEM40 on CC cell proliferation, we further evaluated whether TMEM40 plays a role in CC cell cycle and apoptosis. Cell cycle analysis found that TMEM40 knockdown induced G0/G1 phase arrest, but overexpression of TMEME40 could compensate this for effect in CaSki cells (Fig. 5a–d). Flow cytometry analysis showed a significantly reduced proportion of Annexin V-positive cells in OE-TMEM40 CaSki cells compared to control cells (Fig. 5e–h).

### TMEM40 promoted in vivo tumorigenesis in CC cell lines

**Fig. 6:**
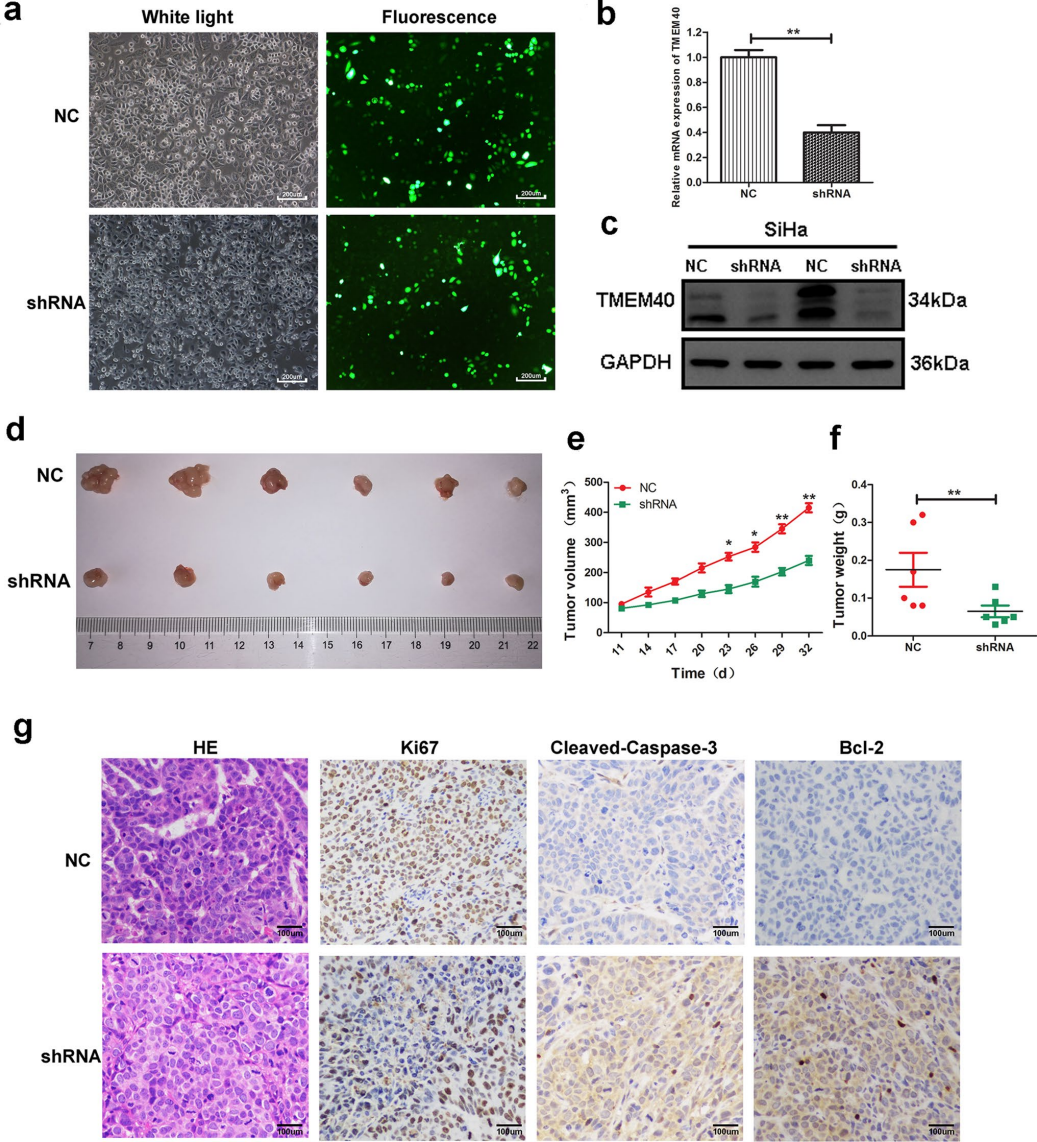
TMEM40 promotes CC cell tumorigenesis in vivo. **a,** The expression of green fluorescent protein after 48 h of TMEM40 shRNA transfection in SiHa cells (× 100). **b** and **c**, Transfection efficiency was tested by western blot and qRT-PCR. **d**, Tumor samples in nude mice induced by SiHa cells transfected with NC or TMEM40 shRNA at 32 days after injection. **e**, Tumor volumes were calculated every 3 days beginning 11 days after injection. **f,** Tumor weights in nude mice at 32 days after injection. **g**, Images of CC samples by H&E staining, or IHC staining for Ki67, Cleaved-Caspase-3 and Bcl-2. (× 200). *P < 0.05, **P < 0.01

To further investigate the in vivo tumor inhibition effect of TMEM40 knockdown, we subcutaneously injected SiHa cells transfected with TMEM40 shRNA and shNC plasmid (Fig. 6a–c) in nude mice to establish the xenograft models. The nude mice were randomly divided into two groups. After 4 weeks of modeling, tumor size and weight were significantly lower in the shRNA TMEM40 group than shNC group (Fig. 6d–f). In addition, the tumor growth rate of TMEM40 shRNA group was distinctly slower than that of shNC group (Fig. 6e). Immunohistochemistry analyses further uncovered significant increments of Cleaved-caspase-3 and Bcl-2 positive cells in the TMEM40 shRNA xenografts compared with controls. Furthermore, our results found that Ki-67 expression in tumors in the shNC group was more than that in the TMEM40 shRNA group, and HE staining showed consistent results (Fig. 6g). These results suggested that down-regulating TMEM40 expression can inhibit the proliferation of tumor cells in vivo.

### TMEM40 acted as an oncogene via regulating the p53 pathway

**Fig. 7:**
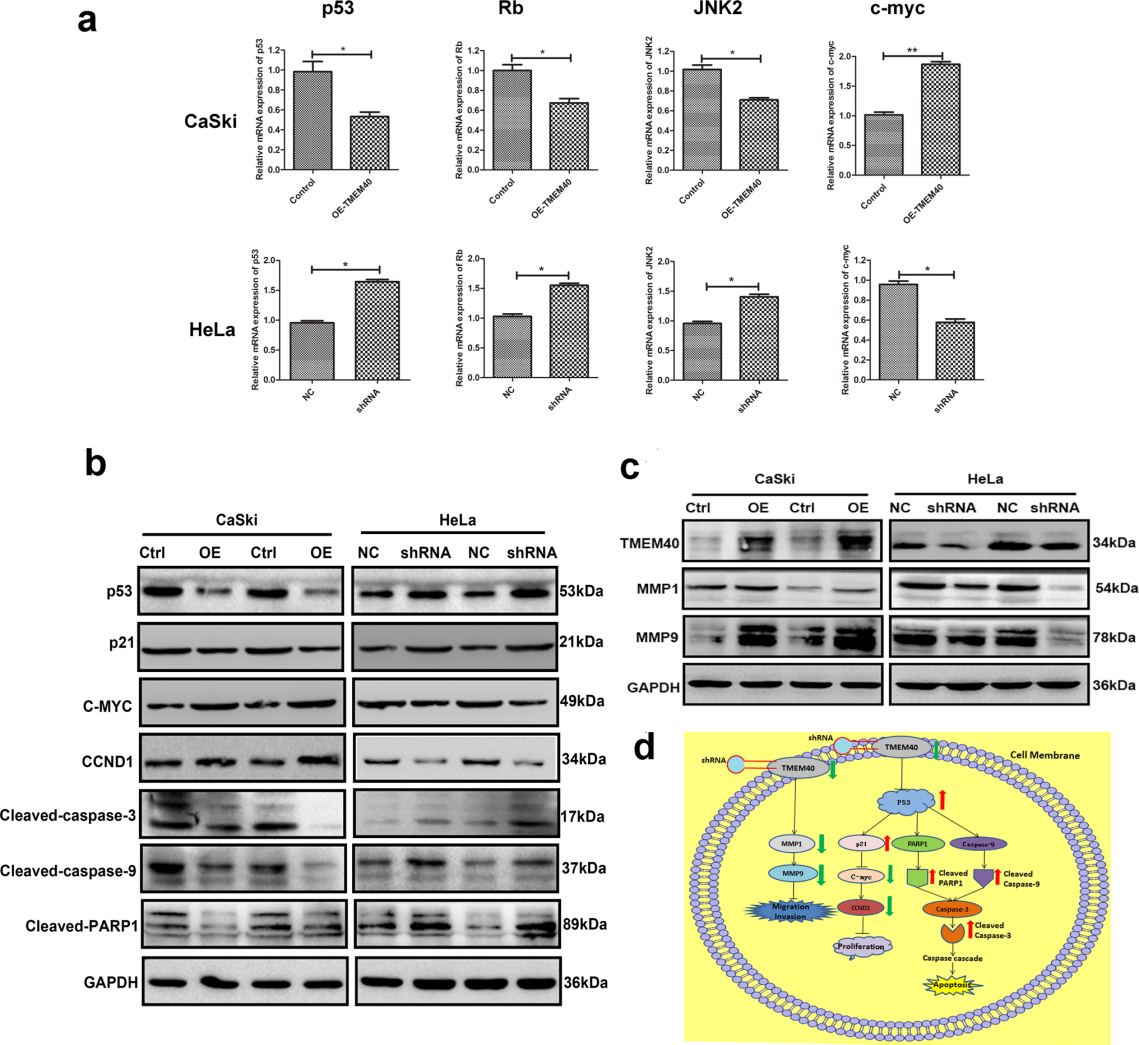
TMEM40 acts as an oncogene via regulating the different signaling pathways. **a**, Quantitative RT-PCR of c-MYC, p53, Rb and JNK2 in CaSki and HeLa cells. **b** and **c** Western blot analysis of proliferation, invasion, apoptosis related signals in CC cells. **d** The working model of TMEM40-regulated CC progression and development. Data are shown as mean ± SD. *P < 0.05, **P < 0.01

To further demonstrate that TMEM40 is an oncogene that promotes tumorigenesis, we detected the expression levels of c-MYC as well as p53, Rb, and JNK2 in shNC cells and TMEM40 shRNA-transfected cells, which have been reported to be widely involved in epithelial tissue tumorigenesis. TMEM40 overexpression resulted in significant downregulation the expression of suppressor genes p53, Rb and JNK2 as well as significant upregulation the expression of oncogenes c-MYC (Fig. 7a). To further study the signaling mechanism of TMEM40 regulating CC cell proliferation, invasion and apoptosis. we detected the change of proliferation, invasion-related signals (i.e. p53, p21, c-MYC, cyclinD1, MMP1, MMP9) and apoptosis-related proteins (i.e. p53, activated caspase-3, activated caspase-9, activated PARP) by western blot. Knockdown of TMEM40 increased the expression levels of p53 and p21, activated caspase-3, caspase-9 and PRAP, and decreased the levels of c-MYC, cyclinD1, MMP1 and MMP9 in HeLa cells (Fig. 7b, c). Taken together, these results suggested that TMEM40 knockdown significantly inhibited CC cell proliferation, invasion and promoted apoptosis (Fig. 7d).

## Discussion

As a common gynecological malignancy, CC is a great threat to women’s health, especially in developing countries [12, 13]. Although CC can be prevented by vaccination against the HPV virus, there are still many limitations to vaccination. So there is an urgent need to explore new therapeutic targets facing CC. It is well known that increased cell proliferation and invasion and decreased apoptosis are the characteristics of tumor malignant progression [14]. Therefore, searching for molecular regulators of tumor cell growth has been an important goal of cervical cancer research. TMEM family proteins can span the entire lipid bilayer and anchor to the membrane [15]. Recently, it has been proved that the TMEM family is involved in the regulation of cervical cancer, in which TMEM48 promotes cell proliferation and invasion by activating the Wnt/-βcatenin pathway [16, 17]. Recent study have shown that serum TMEM40 has good diagnostic value, which is helpful to improve the early diagnosis of human hepatocellular carcinoma [18]. Meanwhile, the regulation of TMEM40 on malignant behavior of other tumors has been reported [10, 12, 19, 20]. These studies suggested that TMEM40 may be an attractive molecule for the diagnosis and treatment of tumors. In this study, we explored the potential function of TMEM40 in CC and confirmed that TMEM40 promotes the progression of CC by down-regulating the expression of p53, Rb, and JNK2, and up-regulating the expression of oncogene c-MYC.

In the present study, through the analysis of GEPIA website, we learned that TMEM40 is markedly upregulated in CC tissues. We further validated in 15 matched human CC and normal tissue by western blot and  quantitative RT-PCR. We also verified the expression of TMEM40 in CC cell lines with different pathologic subtypes. Subsequently, we selected CaSki and HeLa cells to overexpress or knockdown TMEM40 in vitro to observe its effect on tumor progression. As expected, overexpression of TMEM40 promoted cell proliferation, migration, and invasive activity. Conversely, after knocking down TMEM40 the result was inhibition in HeLa cells. Consistently, a study demonstrated that low expression of TMEM40 was associated with better OS in CC, which supported the results of our experiment [11]. The main goal of clinical cancer therapy has been to develop therapies that promote the effective elimination of cancer cells through apoptosis. Therefore, it is profound clinical significance to search for potential targets that can promote tumor cell apoptosis [21]. So, we continued to explore whether TMEM40 plays a key role in regulating cell cycle and apoptosis. Surprisingly, repressed expression of  TMEM40 blocked the transformation of G1-to-S cell cycle and inhibited cell apoptosis. These effects could be reversed by overexpressing it in CaSki cell. Furthermore, we verified the effect of TMEM40 on CC progression in vivo, and the results showed that silencing TMEM40 could dramatically inhibit tumorigenesis and growth. All these results emphasized that the potential clinical significance of TMEM40, which prompted us to further explore the possible mechanism of TMEM40 regulation in CC.

At present, little is known about the mechanism of TMEM40 in tumor progression. Cyclins and CDKs regulate G1 phase progression of the cell cycle. The release of G1/S phase transition is an essential step in the development of CC [22–24]. In this study, we found that TMEM40 can regulate the G1-S phase transition process, thereby enhancing cell proliferation. Because of the complex mechanisms of tumorigenesis, we examined the classical signaling pathways (c-MYC, p53, Rb, and JNK2) in tumors when TMEM40 expression changed. Our results showed that TMEM40 knockdown increased the levels of p53 and p21, activate caspase-3, caspase-9 and PRAP, and decreased the levels of c-MYC and cyclinD1 in HeLa cells. Overexpression of MMP1 and MMP9 in solid tumors was associated with tumor invasion and metastasis have been reported [25–27]. We finally illustrated that TMEM40 could activate the MMP1/MMP9 pathway during the progression of CC. These results indicated that TMEM40 is involved in the regulation of CC via multiple signaling pathways as an important regulator of CC. However, the multiple signaling pathways underlying this regulatory effect remain to be elucidated in future studies.

## Conclusions

In summary, the present study demonstrated that TMEM40 was upregulated in CC tissues and cell lines, the enhanced expression of TMEM40 is associated with lymph node metastasis and poor prognosis. Functional studies suggest that TMEM40 promotes tumorigenesis in CC. Mechanically, TMEM40 downregulated the expression of suppressor genes p53, Rb and JNK2 as well as effected the change of proliferation, invasion-related gene, and then leading to tumor growth (Fig. 7d). However, the detailed mechanism of function of TMEM40 remains to be further studied. These results suggest that TMEM40 is a novel target and molecular therapeutic strategy for CC and reveal the role of TMEM40 in the progression of CC.

## Data Availability

The datasets used and/or analyzed during the current study are available from the corresponding author on reasonable request.
